# Start With the End: Early Hospital Discharge Planning as a Day-One Priority

**DOI:** 10.7759/cureus.87019

**Published:** 2025-06-30

**Authors:** George Bechir, Angelina Bechir

**Affiliations:** 1 Hospital Medicine, Franciscan Health, Munster, USA; 2 Department of Genetics and Biochemistry, Clemson University, Clemson, USA

**Keywords:** care transitions, discharge coordination, early discharge planning, estimated discharge date, family engagement, hospital communication, hospital throughput, multidisciplinary rounds, patient-centered care, whiteboard communication

## Abstract

Despite decades of operational improvement, discharge delays remain a widespread and costly challenge across hospitals. These delays contribute to overcrowded units, inefficient use of resources, and avoidable patient harm. Importantly, many of these delays are not due to unresolved medical issues but to late-stage communication breakdowns, unclear expectations, and inadequate preparation for the transition out of the hospital. Too often, discharge is approached reactively, rather than proactively integrated into the patient’s care plan from the outset. This narrative review reframes discharge planning through a critical but underutilized lens: early and proactive engagement of patients and families. We examine the growing body of evidence supporting the use of tools such as bedside whiteboards, structured interdisciplinary rounds, Estimated Date of Discharge (EDD) documentation, and day-one family contact. Studies show that when the EDD is established within 24 hours and reinforced throughout the stay, hospitals experience fewer non-medical delays, improved patient and caregiver satisfaction, and reductions in length of stay. Furthermore, this approach improves coordination among care teams by providing a shared, visible timeline around which clinical decisions and discharge planning activities can be organized. However, despite its benefits, widespread implementation is hindered by cultural inertia, inconsistent documentation, competing clinical priorities, and a lack of standardized protocols. Drawing on published quality improvement studies and institutional practices, we propose a Day One Discharge Engagement model as a scalable strategy for transforming the discharge process into a continuous, collaborative, and patient-centered effort. This model not only improves hospital efficiency but reframes discharge as an integral part of healing, one that begins at admission and culminates in a safe, supported transition home.

## Introduction and background

Despite advances in hospital operations, discharge delays remain a significant and costly barrier to safe, efficient care. In many institutions, discharge planning still occurs too late in the hospitalization, resulting in missed opportunities to prepare patients and families for safe transitions. This narrative review reframes discharge planning through a powerful yet underused lens: early engagement of patients and families.

In previous works, we highlighted two often-overlooked opportunities for optimizing hospital discharge: the safe discharge of patients with pending microbiology cultures [1] and the impact of a dedicated discharge coordinator on reducing delays and improving patient throughput [2]. Building on these contributions, this review shifts focus to the critical starting point of discharge efficiency: when and how patients and families are engaged. For example, hospitals frequently encounter situations where a patient is medically stable, but discharge is delayed because the family was unaware of the timing and unable to arrange transportation or support at home. Communicating an estimated discharge date from day one allows families to plan ahead, reducing these avoidable delays. To address these challenges, we introduce the Day One Discharge Engagement model, a structured approach that centers discharge planning from the first day of hospitalization.

Hospitals in the United States and abroad continue to face challenges such as non-medical delays, fragmented discharge coordination, and unpredictable lengths of stay, all of which drive up costs and diminish patient satisfaction [3]. While many health systems have adopted tools like discharge planning templates and multidisciplinary rounds, few have implemented these within a framework that explicitly prioritizes discharge from "day one."

Robust evidence supports the practice of establishing and communicating an Estimated Date of Discharge (EDD) within the first 24 hours of admission [3,4,5,6,7]. However, this is not simply a scheduling formality; it reflects a deeper cultural and operational shift. When patients and families are informed of the expected discharge date early in the hospital stay, they are more likely to feel in control, experience smoother transitions, and have adequate time to prepare for post-discharge needs [6,7,8,9]. Communication interventions that incorporate discharge timelines, especially when supported by bedside whiteboards and proactive family engagement, are linked to reduced readmissions, higher satisfaction, and improved transitions of care [8]. Clinical teams also benefit: when the EDD is clearly documented on whiteboards, in the electronic medical record, and discussed during daily rounds, care becomes more coordinated, consults are initiated earlier, and variability in discharge timing decreases [10,11,12].

Hospitals that have introduced whiteboard-centered communication, structured discharge huddles, and resident-led EDD workflows have reported measurable gains in predictability, satisfaction, and care coordination [8,11,13,14,15,16,17]. Patients are not only more aware of the discharge plan but also more likely to take timely action on key steps such as arranging transportation, scheduling follow-up appointments, and obtaining medications before discharge orders are finalized [8,18,19].

This review synthesizes evidence-based strategies and quality improvement efforts from diverse institutional settings. Drawing on current research, implementation protocols, and real-world lessons, we present the Day One Discharge Engagement model: a scalable, team-driven framework that redefines discharge not as an endpoint but as a continuous, visible, and collaborative effort. When consistently applied, this model enhances alignment, reduces waste, and places patients and families, where they belong, at the center of the discharge process.

## Review

Methodology

This narrative review draws on literature published between 2012 and 2025, identified through targeted searches in PubMed, Google Scholar, and institutional repositories using terms such as "early discharge planning," "estimated discharge date (EDD)," "multidisciplinary rounds," "bedside whiteboards," and "hospital throughput." Sources included peer-reviewed articles, quality improvement reports, and institutional protocols. We included English-language documents that described discharge-related interventions, measured outcomes such as length of stay or patient satisfaction, or provided practical implementation frameworks. Opinion pieces without intervention details or data were excluded. Findings were synthesized thematically to highlight evidence-based practices and common barriers to early discharge engagement.

As a narrative review, article selection was based on relevance to the topic, and we acknowledge the potential for selection bias due to the lack of formal inclusion criteria. Themes were developed inductively during the review process based on recurring patterns across sources. No formal quality appraisal was performed, as the review prioritized practical relevance and real-world implementation insights.

Why early engagement matters

Hospital discharge is more than a final act; it is a process that unfolds throughout the hospitalization. When patients and families are engaged from the beginning, they gain a clearer understanding of what is needed for a safe transition and are better equipped to participate in the plan of care. This engagement is not only a courtesy; it is a powerful driver of improved outcomes.

Reduces Length of Stay (LOS)

Early communication of the EDD enables all stakeholders, including patients, families, nurses, case managers, and consultants, to align around a shared timeline. Setting the EDD within 24 hours prompts proactive planning, facilitates timely consults, and minimizes unnecessary hospital days [6-8]. Institutions that implement EDD workflows report measurable LOS reductions, especially when the EDD is revisited daily during interdisciplinary rounds [13,14,18].

Improves Patient Satisfaction

Patients who are informed early about anticipated discharge experience less uncertainty and greater confidence in their care. Displaying the EDD on bedside whiteboards reinforces this message and has been associated with higher satisfaction scores [13,14]. Families benefit as well, gaining time to arrange transportation, home support, or post-acute care logistics [10,15].

Enhances Safety and Readiness

Initiating discharge discussions early allows sufficient time for education, follow-up planning, and medication review. Studies show that proactive family involvement improves adherence to discharge instructions, reduces 30-day readmissions, and supports smoother transitions to home or facility-based care [9,12]. The AHRQ IDEAL Discharge framework recommends ongoing patient and caregiver education throughout hospitalization [6].

Strengthens Team Coordination

When the EDD is visible and actively discussed, it becomes a unifying objective for the care team. This shared timeline helps team members prioritize discharge-related tasks more effectively [13,14]. Leading hospitals require the EDD to be documented early and reviewed in multidisciplinary rounds to promote alignment [16,18,19].

Addresses Non-Medical Delays

Even when medical goals are met, discharge may be postponed due to unresolved social, logistical, or emotional issues. Early family contact helps identify and address these barriers in advance. International studies show that delayed or insufficient discharge communication contributes to patient anxiety and poor transitions [20,21]. Proactive planning ensures that psychological and logistical readiness keep pace with clinical recovery [6,21].

Strategies and tools that support early engagement

While the rationale for early discharge engagement is well established, successful implementation depends on practical strategies that make discharge planning visible, collaborative, and patient-centered from the first hospital day.

Set the EDD Within 24 Hours

Among available tools, few are as impactful as early EDD entry. Institutions that standardize EDD settings within 24 hours of admission report stronger team alignment, earlier specialist consults, and fewer day-of-discharge barriers [6,7,8]. Although the EDD may change, initiating a target date creates momentum and shared accountability.

Use Bedside Whiteboards as a Communication Anchor

Bedside whiteboards, though low-tech, consistently improve transparency. When the EDD is clearly posted at the bedside, patients better understand their discharge plan, and care teams report greater goal alignment [13,14,17,18]. Hospitals using structured whiteboard templates, with fields for EDD, goals, and family contacts, standardize messaging and reduce confusion.

Conduct Daily Interdisciplinary Discharge Rounds

Multidisciplinary rounds (MDRs) offer a reliable venue for reviewing discharge readiness and identifying obstacles early. When rounds include nurses, case managers, therapists, pharmacists, and physicians, with explicit EDD discussion, lengths of stay decrease and discharges occur more predictably [16,19,21]. MDR also facilitates timely referrals and discharge support services.

Make First-Day Family Contact a Standard

Early outreach to the patient's support system allows for more effective planning. Even a brief introductory call on day one gives families a chance to ask questions, voice concerns, and prepare for discharge needs. Standardizing this outreach helps identify social or logistical challenges well in advance [10,11,20].

Track and Audit EDD Accuracy

EDD use is only meaningful when monitored for consistency and reliability. Hospitals that audit EDD changes, accuracy rates, and reasons for delays can identify process gaps-such as rehab placement bottlenecks or consultant lag times-and take corrective action [19]. Resident-led tracking tools and EMR dashboards have improved compliance in academic settings.

Integrate EDD Into Electronic Health Records and Care Boards

When EDD information is embedded in rounding dashboards, patient lists, and care boards, team members across disciplines-including consultants and ancillary staff-can act with better coordination [13,20]. EDD visibility also supports discharge checklists, readiness scoring, and follow-up planning tools that integrate into routine workflows.

Barriers to implementation

Despite strong evidence and low-cost tools, the widespread adoption of early discharge engagement remains limited. Understanding the operational and cultural challenges is essential to designing sustainable solutions.

Cultural Norms That Treat Discharge as a Final Step

In many hospitals, discharge planning begins only after clinical stabilization, reinforcing the idea that discharge is an endpoint rather than an ongoing process. Physicians may hesitate to set an EDD early, concerned about changing clinical conditions. Addressing this requires deliberate leadership messaging, resident education, and integration of discharge planning expectations into rounding workflows. Institutions that normalize EDD documentation on day one, as part of admission notes or consultant evaluations, can shift this mindset through repetition and accountability.

Lack of Standardized Protocols and Accountability

When EDD entry is optional or unmonitored, practice varies widely. Clear policies that mandate EDD entry within 24 hours, coupled with weekly audits and performance dashboards, have been used successfully in academic and private health systems [19]. Embedding EDD fields into EMR templates and requiring updates during interdisciplinary rounds ensures the plan remains current and visible.

Communication Gaps Within the Care Team

Even with an EDD in place, a lack of cross-disciplinary communication can undermine its effectiveness. Nurses, therapists, and consultants may be unaware of each other’s plans, leading to misalignment. Hospitals that adopt structured whiteboard templates and standardized multidisciplinary rounds (including review of EDD, barriers, and disposition plans) have reported better coordination and fewer last-minute surprises [13,16,18].

Failure to Consistently Involve Families

Engaging families on day one requires time and a clear trigger for action. Creating a standard that prompts day-one family outreach, such as a script for case managers or nursing staff, helps institutionalize this step. Including family contact info and readiness concerns directly on bedside whiteboards or discharge checklists further reinforces this practice.

Limited IT Infrastructure or Workflow Integration

When EDDs are not visible on daily worklists, care boards, or rounding dashboards, they are often overlooked. Ensuring EDD fields are built into electronic rounding tools - and connected to discharge readiness scoring systems - can make discharge planning part of the team’s daily rhythm. IT departments should collaborate with clinical leads to ensure display fields are optimized for workflow, not buried deep in documentation. Standard operating protocols and implementation guides, such as those described in recent systematic reviews of discharge communication strategies [22], provide useful models for how to embed these tools into daily practice. Implementation strategies are embedded throughout the review to align with clinical workflows and avoid redundancy.

The Day One Discharge Engagement model

The Day One Discharge Engagement model is a structured, team-based framework that reimagines hospital discharge as a process that begins at admission, not a task reserved for the final day. It integrates early communication, interdisciplinary coordination, and visible planning, with a strong emphasis on engaging the patient and family from the outset to ensure alignment, expectation-setting, and readiness for a safe transition home.

Rather than relying on reactive, last-minute steps, this model embeds discharge planning into the daily workflow of every provider, starting within the first 24 hours. Clinical decisions, therapy goals, case management, and patient education are all aligned around a shared discharge target. Patients and families are treated not as passive recipients of information, but as active partners in the plan.

The model includes six key components:

EDD Set on Day 1

The EDD is established within the first 24 hours of admission. This creates a shared target that promotes early team alignment and initiates the discharge planning process. Even if the date evolves, having a goal from the start helps build momentum and accountability.

Visual Documentation of the EDD

The EDD is recorded in the EMR and posted on the patient’s bedside whiteboard. This keeps the plan visible not only to the care team but also to the patient and their family, reinforcing daily awareness of discharge goals and timelines.

Daily Interdisciplinary Discharge Rounds

Structured discharge rounds include physicians, nurses, case managers, pharmacists, therapists, and other key staff. These rounds review the EDD, assess barriers, and update the plan. When possible, patients and families are informed of progress and involved in clarifying next steps.

Day One Family and Patient Engagement

The patient's family or primary caregiver is contacted on day one to discuss the anticipated discharge plan, confirm support needs, and identify any barriers early, such as transportation, home environment, or follow-up care logistics. Just as importantly, the patient is engaged in the discussion of their expected discharge timeline, what milestones must be met, and what role they play in preparation. Setting expectations early helps reduce anxiety, align goals, and create a sense of shared responsibility. Regular updates ensure patients and families stay informed and empowered throughout the hospitalization.

Tracking EDD Accuracy and Delays

Hospitals monitor whether the EDD is met, changed, or delayed and why. This promotes learning across the system, highlights bottlenecks (e.g., rehab placement, consult delays), and supports targeted process improvements.

Workflow Integration into EMR and Team Tools

Discharge planning is integrated into daily notes, care boards, rounding templates, and team checklists. This makes the discharge timeline a daily priority and ensures that all care activities, including education, medications, and follow-up, stay aligned with the patient’s expected transition date.

**Figure 1:**
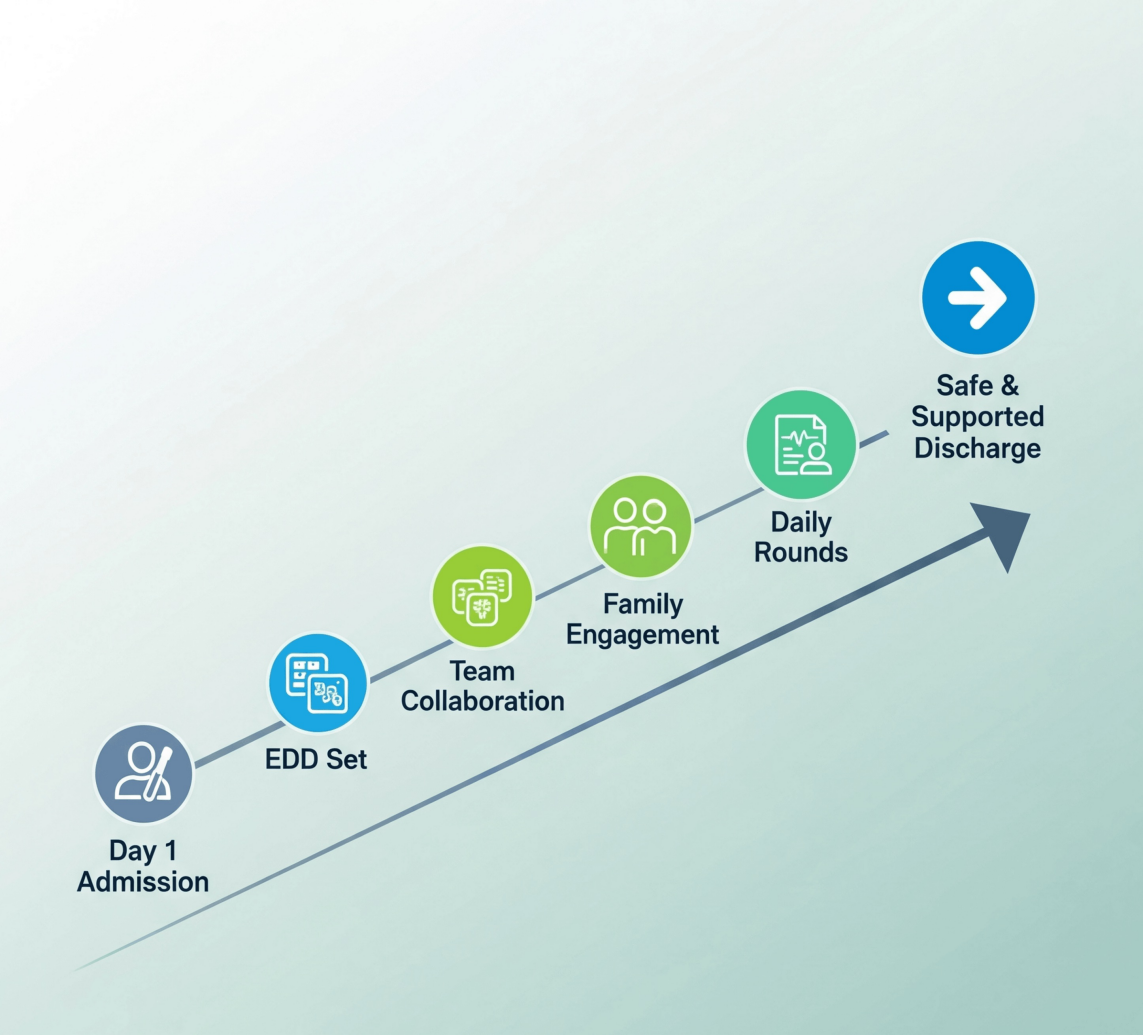
The Day One Discharge Engagement model: a structured, six-step framework for aligning teams, patients, and families around discharge from the first day of hospitalization.

This model transforms discharge into a visible, continuous, and collaborative process. By including the patient and family from day one, the care team creates a more predictable, respectful, and well-coordinated experience. Figure 1 illustrates this stepwise approach from admission to safe, supported discharge.

## Conclusions

Early engagement of patients and families in discharge planning is a proven strategy that remains underutilized in many hospital settings. While complex medical and post-acute needs do contribute to prolonged hospitalizations, a substantial number of delays are preventable, often caused by breakdowns in communication, lack of role clarity, or uncoordinated transitions. As outlined in this review, setting and sharing the EDD within the first 24 hours - reinforced through visual tools, structured rounds, and proactive family contact - can improve readiness, shorten length of stay, and elevate the overall quality of care. However, these interventions are only effective when embedded into routine workflows, supported by leadership, and practiced consistently across teams. Building on our earlier work on microbiology clearance and discharge coordination, we propose a Day One Discharge Engagement model that redefines discharge not as a late-stage task but as a shared, continuous responsibility from admission forward. Hospitals that adopt this approach can improve patient flow, reduce costs, and deliver care that is more transparent, respectful, and patient-centered. Discharge is not a transaction-it is a transition. Preparing for it should begin on day one.

While this review synthesizes practical and evidence-informed strategies, several limitations should be acknowledged. Many of the cited studies reflect single-center quality improvement efforts, which may limit generalizability to other settings. In addition, as a narrative review, this work does not include a systematic appraisal of study quality or risk of bias. Relevant implementation details or unpublished interventions may have been missed due to limitations in available literature. Finally, some promising strategies, such as EDD documentation or bedside whiteboards, are supported by emerging evidence that warrants further validation through larger, controlled studies. To advance the field, future research should focus on evaluating the Day One Discharge Engagement model across varied hospital settings and patient populations. Prospective studies, multicenter implementation trials, and comparative effectiveness analyses are needed to assess outcomes such as discharge timing accuracy, length of stay, readmission rates, and team communication metrics. In addition, studies should explore how to adapt this model in resource-limited environments and determine which components have the greatest impact when implemented individually versus in combination. Establishing standardized benchmarks for discharge readiness, EDD adherence, and family involvement would also strengthen ongoing quality improvement efforts.
